# Deciphering the role of Ki67: prognostic insights in advanced breast cancer therapy with Cyclin-dependent kinase 4/6 inhibitors

**DOI:** 10.1186/s12885-026-15594-2

**Published:** 2026-02-13

**Authors:** Marcin Kubeczko, Anna Polakiewicz-Gilowska, Katarzyna Świderska, Marta Mianowska-Malec, Aleksandra Leśniak, Barbarba Łanoszka, Konstanty Chomik, Barbara Grandys, Natalya Lisovska, Grzegorz Woźniak, Tomasz Latusek, Ewa Stobiecka, Jakub Simek, Bartłomiej Pyciński, Barbara Bobek-Billewicz, Ewa Chmielik, Michał Jarząb

**Affiliations:** 1 Breast Cancer Center, Maria Sklodowska-Curie National Research Institute of Oncology Gliwice Branch, 15 Wybrzeze Armii Krajowej Street, Upper Silesia, 44-102 Gliwice, Poland; 2 Radiotherapy Department, Maria Sklodowska-Curie National Research Institute of Oncology, Gliwice, 44-102 Poland; 3 Tumor Pathology Department, Maria Sklodowska-Curie National Research Institute of Oncology, Gliwice, 44-102 Poland; 4 Faculty of Biomedical Engineering, Silesian University of Technology, Zabrze, 41- 800 Poland; 5 Radiology and Diagnostic Imaging Department, Maria Sklodowska-Curie National Research Institute of Oncology, Gliwice, 44-102 Poland

**Keywords:** Advanced breast cancer, Ki67, Ribociclib, Abemaciclib, Palbociclib

## Abstract

**Background:**

Cyclin-dependent kinase 4/6 inhibitors (CDK4/6i) are the standard of care in advanced breast cancer. While Ki67 is prognostic in early-stage disease, including adjuvant CDK4/6i, its role in metastatic setting is unclear. This study assessed Ki67’s prognostic value in advanced breast cancer treated with CDK4/6i.

**Methods:**

We conducted a retrospective analysis of patients with advanced breast cancer receiving CDK4/6i at our cancer center between 2018 and 2023.

**Results:**

The study included 368 patients with Ki67 results. The median age was 63 years (IQR 52–70.5). 283 patients (76.9%) received CDK4/6i in the first-line. With respect to biopsy timing, Ki67 expression was evaluated in tissue samples from 319 patients in the metastatic setting and 49 patients in the early breast cancer setting. In terms of biopsy site, Ki67 was measured from the breast tumor in 235 patients, from regional lymph nodes in 26 patients, and from various metastatic sites in 107 patients. Ki67, as a continuous variable, correlated with PFS and OS. Specifically, each unit increase in Ki67 was associated with a 1% reduction in PFS and OS. Median PFS in patients with lowKi67 was 33.0 months vs. 22.0 months for highKi67 (*p* = 0.008). Two-year PFS was 68.3% for lowKi67, vs. 48.4% for highKi67. Median OS was 49.7 months for lowKi67 vs. 39.8 months for highKi67 (*p* = 0.027).

**Conclusions:**

Ki67 appears to be a prognostic factor in advanced breast cancer patients treated with CDK4/6i. Patients with low Ki67 had favorable prognosis.

**Supplementary Information:**

The online version contains supplementary material available at 10.1186/s12885-026-15594-2.

## Introduction

 Breast cancer remains the most commonly diagnosed malignancy worldwide [1]. In early-stage breast cancer, the standard assessment at diagnosis includes determining immunohistochemistry (IHC) evaluation of key biomarkers: estrogen receptor (ER), progesterone receptor (PgR), and human epidermal growth factor receptor 2 (HER2), and a proliferation marker Ki67 [2]. IHC profiling enables the categorization of tumors into surrogate intrinsic subtypes according to the St Gallen Consensus guidelines [3]. The assessment of Ki67, a pivotal proliferation marker, should be guided by the recommendations of the International Ki67 in Breast Cancer Working Group [4].

Ki67 plays an important role in early breast cancer management. A decrease in Ki67 (≤ 10%) after short-term (4–6 weeks) preoperative endocrine therapy (ET) in postmenopausal patients with HR-positive, HER2-negative breast cancer is a prognostic and indicates endocrine responsiveness [5, 6].

Cyclin-dependent kinase 4/6 inhibitors (CDK4/6i) have emerged as the benchmark treatment for hormone receptor (HR) - positive HER2 - negative advanced breast cancer [7, 8]. Adding CDK4/6i to endocrine treatment has been shown to confer significant progression-free survival (PFS) (9–13) and overall survival (OS) advantages [9–11]. Additionally, their role in early breast cancer is rising. Abemaciclib was the first CDK4/6i approved in the adjuvant setting [12]. Recently, the results of the NATALEE trial were published [13]. Ribociclib significantly improved invasive disease-free survival among patients with HR-positive, HER2-negative stage II or III early breast cancer [13].

High Ki67 was an important factor for the adjuvant treatment with abemaciclib [14]. Nonetheless, the role of Ki67 role in metastatic breast cancer remains a matter of controversy [15]. Thus, we aimed to assess the prognostic value of Ki67 in advanced breast cancer patients treated with CDK4/6i.

## Methods

### Study population

We retrospectively analyzed patients with advanced breast cancer treated in our cancer center with cyclin-dependent kinase 4/6 inhibitors between 2018 and 2023. The study was conducted following the Helsinki Declaration of 1975, revised in 2000, and approved by the bioethics committee in our institution.

The key inclusion criteria comprised: (1) histologically confirmed HR-positive HER2-negative breast cancer, (2) advanced disease, (3) Ki67 assessment, (4) CDK4/6i treatment, and (5) assessment of response.

The study’s primary endpoint was Ki67 association, as a continuous variable, with PFS. The secondary endpoints were: Ki67 association, as a continuous variable, with OS; and the association of Ki67 with PFS and OS using a calculated cutoff.

### Ki67 assay

Immunostaining was performed with the Dako Omnis system and the EnVision FLEX Kit (Dako, Agilent Technologies, Santa Clara, CA, USA) with anti-Ki67 (clone MIB-1, Dako) antibody. Sections were automatically counterstained with hematoxylin (Dako) for 5 min. External positive and negative controls were always used for each assay. Biomarker positivity was detected and semiquantitatively quantified as the percentage between immunopositive tumor cells and the total number of tumor cells.

Our Tumor Pathology Department has undergone external quality control conducted by the Nordic Immunohistochemical Quality Control (NordiQC) assessment of Ki67. The evaluation was based on staining intensity and distribution in cells expected to be demonstrated, background staining, cross-reactivity, counterstaining quality, and tissue morphology preservation. After the assessment, our department received the highest score—optimal—which refers to a staining reaction considered perfect or close to perfect in all of the included tissues.

As part of the standard clinical workflow, nearly all histopathological results from external laboratories — including Ki67 — are routinely re-evaluated at our institution when the submitted tissue material is available and suitable for review. Given our status as the national reference center for oncology, the outcome of this internal re-assessment is considered definitive and takes precedence over any prior external reports.

### Endocrine resistance definitions

Endocrine resistance was defined following the ABC guidelines [7]. Primary endocrine resistance was characterized by relapse while on adjuvant endocrine treatment within the first 2 years or disease progression within the initial six months of first-line endocrine therapy for advanced breast cancer. Secondary endocrine resistance, on the other hand, was defined as relapse while on adjuvant endocrine therapy but after the first two years, relapse within 12 months of completing adjuvant endocrine therapy, or disease progression occurring ≥ 6 months after initiating endocrine treatment for advanced breast cancer.

### Statistical analysis

Categorical variables were presented as frequencies and percentages, while continuous variables were displayed as mean or median values with interquartile ranges (25% to 75%, IQR). PFS and OS were assessed using the Kaplan-Meier method with 95% confidence intervals (95% CI) and log-rank testing. Cox proportional hazard models assessed the effect on PFS and OS. We used the Wilcoxon rank sum test, Fisher exact test, or Chi-square test to assess differences in clinicopathologic parameters between groups of patients with low Ki67 and high Ki67. Subgroup analyses were conducted to explore whether the association between selected clinical characteristics and 24-month PFS or OS differed according to Ki67 expression level. Patients were stratified by Ki67 status (low vs. high), and within each stratum, multivariable logistic regression models were fitted for each variable of interest, adjusting for relevant covariates. Adjusted odds ratios (ORs) were estimated to quantify the association between each clinical variable and outcome risk. High ER and high PR were defined as expression levels equal to or above the median value. Interaction tests were performed using logistic regression models including an interaction term between Ki67 and the clinical variable of interest. Forest plots were generated to visualize stratum-specific effects for both PFS and OS.

All tests were two-sided, with a p-value < 0.05 considered statistically significant. All analyses were conducted with Stata Statistical software (version 18, StataCorp, College Station, TX, USA).

### Ki67 cutoff calculation using the Youden index

The median PFS for patients treated with CDK4/6i in pivotal trials was 24.8 months [16], 28.2 months [17], 25.3 months [18], 20.5 months [19], and 23.8 months [20]. Thus, a timepoint of 24 months progression-free survival was used for Ki67 cutoff calculations.

Ki67 optimal cutoff value was calculated using the Receiver Operator Characteristics (ROC) curve with the Youden index. Ki67 cutoff of > 15% yielded a sensitivity of 0.67, and a specificity of 0.52, with an area under the ROC curve of 0.60. Thus, patients were characterized as having low Ki67 if Ki67 was equal to or less than 15%, whereas Ki67 of more than 15% was characterized as high Ki67. However, we have to emphasize that cutoffs of 20% and 14% operated in quite similar results (area under the ROC curve of 0.56 for Ki14% cutoff, and 0.59 for Ki20% cutoff, respectively).

## Results

### Patients’ characteristics

The study encompassed 368 patients, with a median age of 63 years (IQR 52–70.5). Performance status (PS), as assessed by Eastern Cooperative Oncology Group (ECOG), was distributed as follows: 154 patients had a PS of 0 (41.9%), 161 had a PS of 1 (43.7%), and 53 patients had a PS of 2 (14.4%). A diagnosis of de novo metastatic disease was made in 187 patients (50.8%), while 181 patients (49.2%) presented with recurrent disease. Oligometastatic disease was observed in 89 patients (24.2%). One hundred seventy-eight patients (48.4%) had non-visceral disease, while 190 patients (51.6%) had visceral metastases. Regarding CDK4/6i therapy, 211 patients (57.3%) received ribociclib, 101 patients (27.5%) were treated with palbociclib, and 56 patients (15.2%) received abemaciclib. The majority, 283 patients (76.9%), received CDK4/6i as a first-line treatment for advanced breast cancer.

### Ki67 assessment

In 235 patients (63.9%), Ki67 was measured from breast tumors, in 26 patients (7.0%) from regional lymph nodes, and in 107 patients (29.1%) from distant metastatic sites. Notably, the majority of patients (319, 86.7%) had Ki67 assessed from samples derived in the metastatic setting, while a smaller subset (49 patients, 13.3%) had Ki67 evaluated from samples obtained during the early breast cancer setting. No difference was observed between patients with low and high Ki67 regarding biopsy site (*p* = 0.192) and assessment in metastatic or early breast cancer setting (*p* = 0.279). The median time from biopsy to CDK4/6i commencement was 2.3 months (Q1 1.2 months, Q3 10.1 months).

In 290 patients (78.8%), the samples for Ki67 assessment were derived and assessed at our center. Among the remaining 78 patients (21.2%), 51 had their sample pathology assessment verified at our center. Within this subgroup, we observed a discrepancy in Ki67 assessment between the primary result and our verification in 17 samples (33.3%). Specifically, our verification revealed lower Ki67 levels in 7 cases, while in 9 cases, our assessment yielded higher Ki67 values. In one instance, the primary result reported a range between 14% and 20%, whereas our verification indicated a Ki67 result of 15%.

Baseline patient characteristics with a comparison between groups with low Ki67 and high Ki67 are depicted in Table 1. The groups were well-balanced. However, patients in the group with low Ki67 had a significantly higher number of metastases. Progesterone receptor (PgR) positivity of at least 50% was seen in half of the cohort (185 patients, 50.4%). Ki67 was comparable between tumors with PgR ≥ 50% and those with PgR < 50% (median Ki67 20% in both groups; mean Ki67 24.4% vs. 25.5%, respectively, *p* = 0.606).

**Table 1:** Baseline patient characteristics with a comparison between groups with low Ki67 and high Ki67

Characteristics	Low Ki67 [*n* = 159]	High Ki67 [*n* = 209]	*p*
Age, median (IQR)	65 (54–70)	62 (51–71)	0.21
Polymetastatic#	131 (82.4%)	148 (70.8%)	0.014
ECOG 0 1&2	64 (40.3%) 95 (59.7%)	90 (43.1%) 119 (56.9%)	0.596
De novo	81 (50.9%)	106 (50.8%)	1.00
1st line Tx	125 (78.6%)	158 (75.6%)	0.534
ER, median (IQR) mean	100 (90–100) 93.7	100 (90–100) 91.4	0.051
PgR, median (IQR) mean	50 (0–90) 47.3	40 (5–90) 45.9	0.952
Non-visceral disease	81 (50.9%)	97 (46.4%)	0.401
Visceral metastases	78 (49.1%)	112 (53.6%)	
Baseline liver inv.	32 (20.1%)	49 (23.4%)	0.526
Baseline lung inv.	52 (32.7%)	74 (35.4%)	0.658
CDK4/6i			0.837
Ribociclib	90 (56.6%)	121 (57.9%)	
Palbociclib	46 (28.9%)	55 (26.3%)	
Abemaciclib	23 (14.5%)	33 (15.8%)	

*Abbreviations:* *IQR,* – interquartile range; polymetastatic – more than 5 metastases; *ECOG,* – performance status according to Eastern Cooperative Oncology Group; *Tx,* – treatment; *ER,* – estrogen receptor; *PgR,* – progesterone receptor; #polymetastatic disease defined as > 5 metastases

### Baseline prognostic factors

#### Progression-free survival

**Fig. 1:**
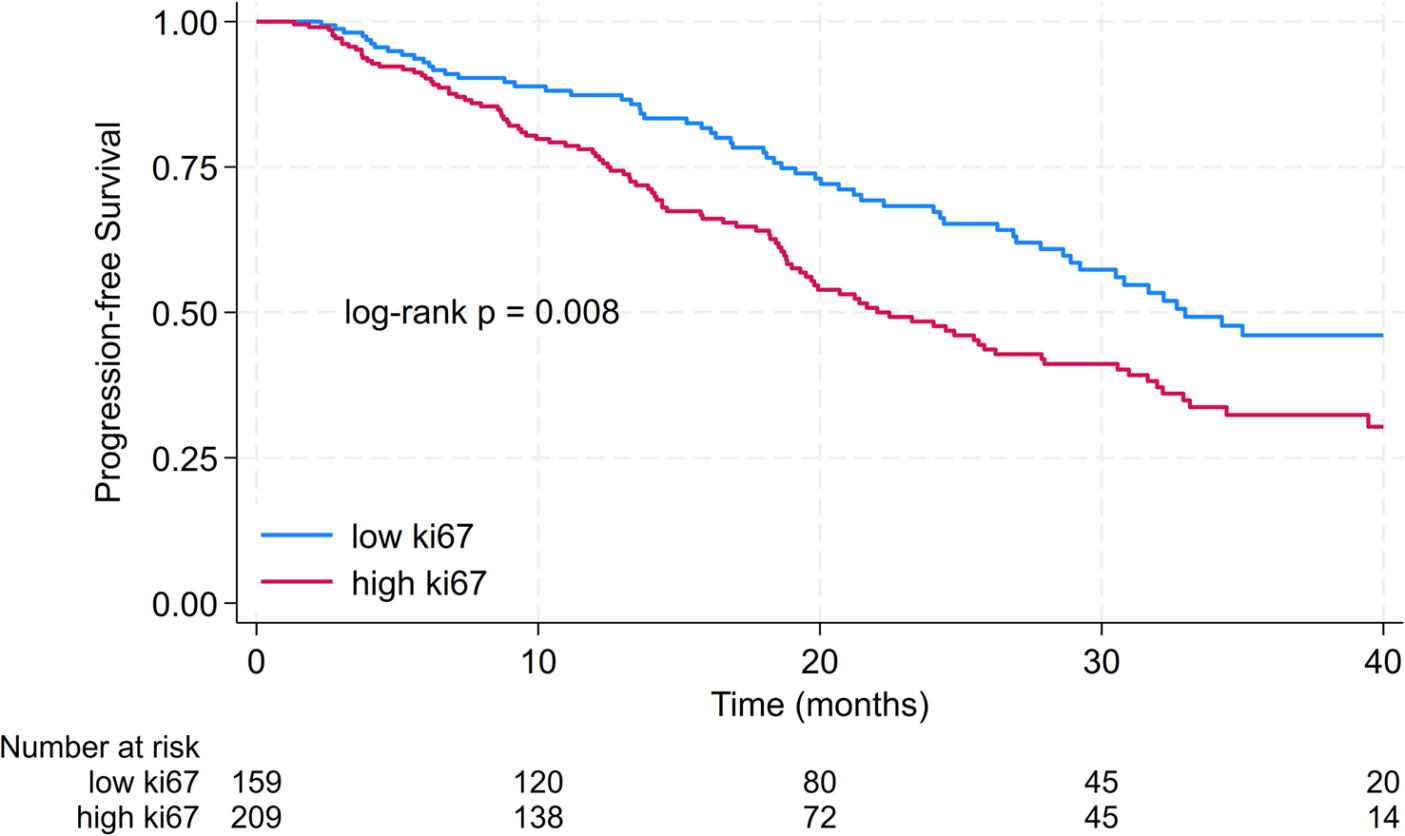
Kaplan-Meier curves for progression-free survival in patients with low ki67 compared to patients with high Ki67

The median PFS was 28.0 months. 2-year PFS was 57.2% [95% CI 51.1–62.8%]. The median PFS in patients with low Ki67 was 33.0 months compared to 22.0 months in patients with high Ki67 (*p* = 0.008). 2-year PFS in patients with low Ki67 was 68.3% [95% CI 59.3–75.7%] compared to 48.4% [95% CI 40.7–56.1%] in patients with high Ki67. Results are displayed in Fig. 1.

Ki67 as a continuous variable was significantly associated with PFS in multivariate analysis. Specifically, each incremental unit increase in Ki67 was associated with a 1% reduction in PFS. Factors contributing to a shorter PFS included polymetastatic disease (defined as more than five metastases), symptomatic disease (characterized by an ECOG PS 1 or 2), presence of metastases in the liver, and endocrine resistance (either primary or secondary). Conversely, higher expression of PgR was associated with extended PFS. The line of treatment, de novo disease, and expression of ER, however, did not remain significant in multivariate analysis. These results are detailed in Table 2. It is also noteworthy that baseline lung/pleural involvement did not prove to be prognostic for PFS (*p* = 0.248) and was consequently not incorporated into multivariate analysis.

**Table 2:** Multivariate Cox regression for progression-free survival

Characteristics		HR*	*p*	95% CI
ECOG	1 & 2 vs. 0	2.00	< 0.001	1.43–2.81
De novo	de novo vs. rec.	0.82	0.275	0.58–1.17
Polymetastatic	> 5 mets vs. less	1.99	0.003	1.27–3.11
Line of Tx	1st vs. 2nd	0.89	0.601	0.59–1.36
ER	continuous	0.99	0.054	0.98-1.00
PgR	continuous	0.99	< 0.001	0.99–0.99
Ki67	continuous	1.01	0.002	1.01–1.02
Baseline liver inv.	yes vs. no	1.65	0.004	1.17–2.31
Endocrine resistance	yes vs. no	1.83	0.005	1.20–2.79

*Abbreviations:* *HR,* – hazard ratio, *ECOG,* – performance status assessed per Eastern Cooperative Oncology Group; rec. – reccurent disease; *Tx,* – treatment; *ER,* – estrogen receptor; *PgR,* – progresterone receptor; inv. – involvement; *HR > 1 indicates a higher hazard, whereas HR < 1 indicates a lower hazard in the first variable category

#### Subgroup Analyses by Ki67 Status for Progression-Free Survival

**Fig. 2:**
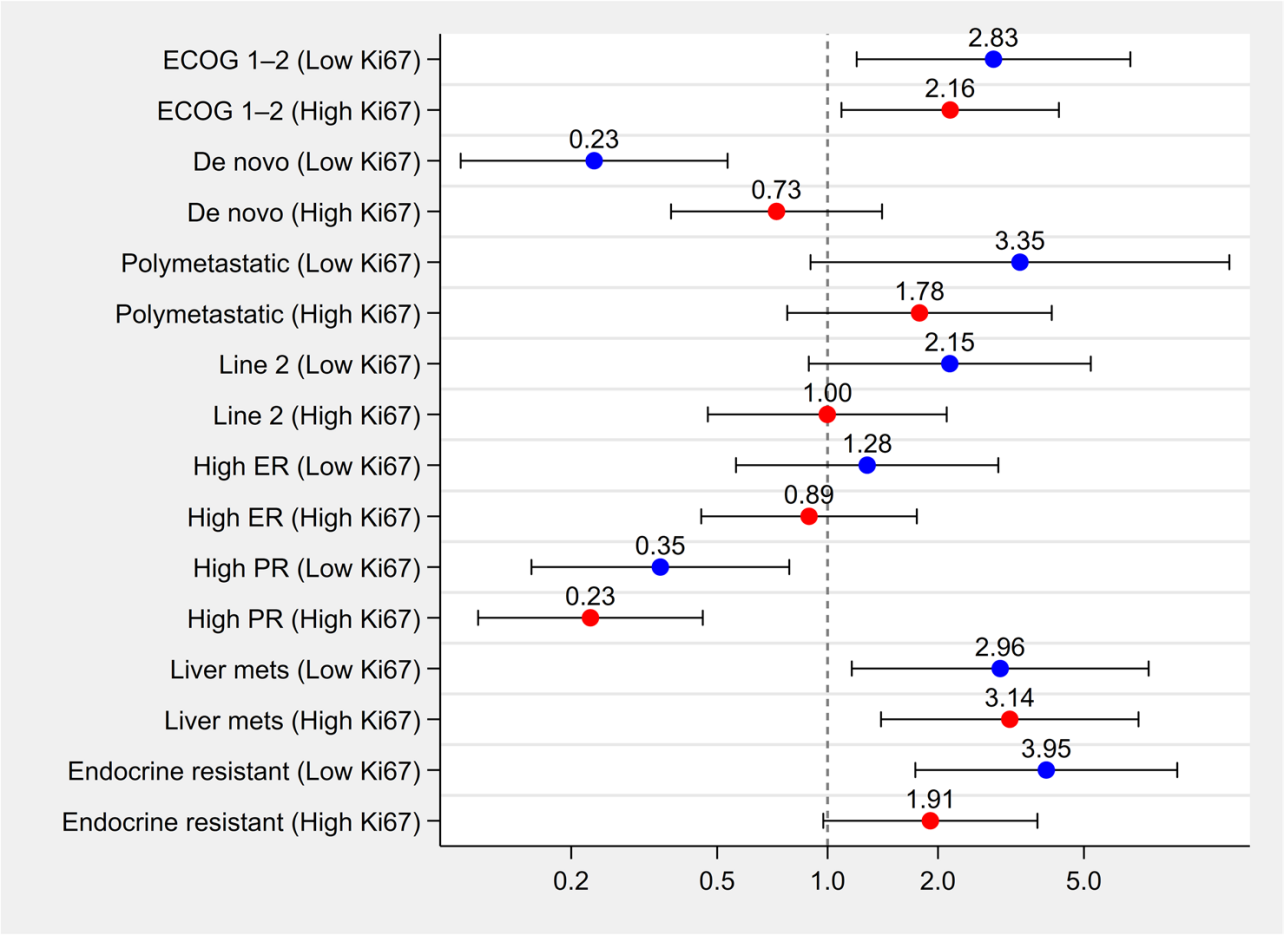
Adjusted odds ratios (ORs) for 24-month overall survival across clinical subgroups, stratified by Ki67 expression level. Each clinical subgroup (e.g., ECOG performance status, disease presentation, metastatic burden, hormone receptor status) was analyzed separately in patients with low versus high Ki67. Multivariable logistic regression models were fitted within each Ki67 stratum. Blue and red dots represent ORs for low and high Ki67 groups, respectively. Horizontal bars indicate 95% confidence intervals. A vertical dashed line at OR = 1.0 marks the null effect

Stratified analyses evaluated whether the prognostic effect of clinical variables differed between low and high Ki67 subgroups (Fig. 2). ECOG 1–2 status was significantly associated with increased risk of progression in both strata (OR = 2.830 for low Ki67; OR = 2.160 for high Ki67; p for interaction = 0.627). De novo presentation was associated with reduced risk in the low Ki67 group (OR = 0.230), but only a modest protective effect in the high Ki67 group (OR = 0.730), yielding a statistically significant interaction (*p* = 0.036). Polymetastatic disease (OR = 3.350 vs. 1.780; *p* = 0.427), second-line treatment (OR = 2.150 vs. 1.000; *p* = 0.194), liver metastases (OR = 2.960 vs. 3.140; *p* = 0.924), and endocrine resistance (OR = 3.950 vs. 1.910; p = 0.179) were all consistently associated with increased progression risk in both strata, though the strength of association varied. Hormone receptor status showed no statistically significant interaction with Ki67. High PR was associated with reduced progression risk across both subgroups (OR = 0.350 for low Ki67; OR = 0.230 for high Ki67; *p* = 0.423), while high ER was not significantly associated with progression in either group (OR = 1.280 vs. 0.890; *p* = 0.502).

Among all tested variables, only de novo vs. recurrent presentation demonstrated a statistically significant interaction with Ki67 expression (*p* = 0.036), suggesting that the prognostic effect of de novo disease may differ depending on tumor proliferative status. For all other variables, interaction *p*-values were non-significant, indicating consistent effects across Ki67 strata.

#### Overall survival

**Fig. 3:**
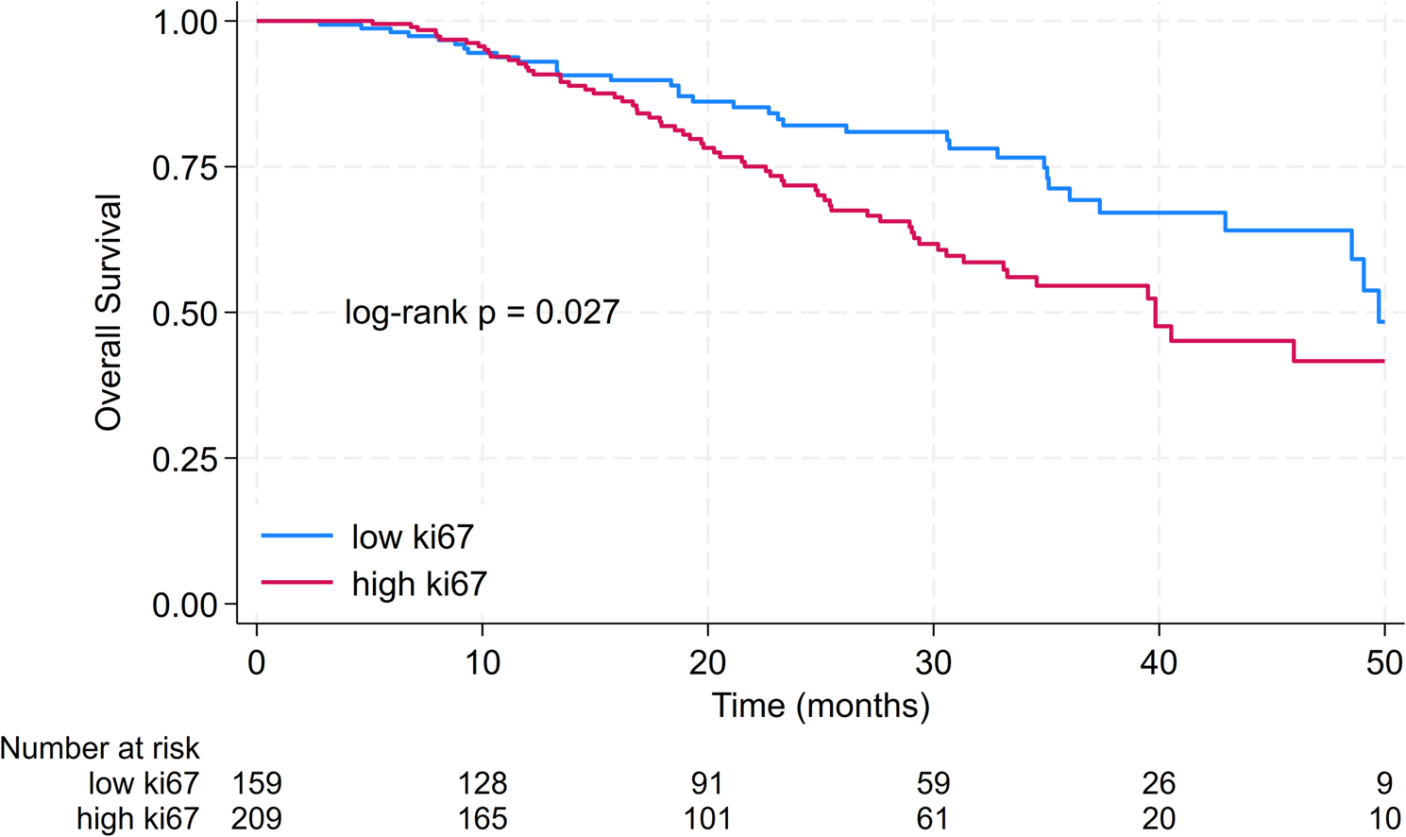
Kaplan-Meier curves for overall survival in patients with low ki67 compared to patients with high Ki67

The median OS was 48.5 months. 3-year OS was 61.9% [95% CI 54.6–68.3%]. The median OS in patients with low Ki67 was 49.7 months compared to 39.8 months in patients with high Ki67 (*p* = 0.027). 3-year OS in patients with low Ki67 was 71.3% [95% CI 60.3–79.7%] compared to 54.6% [95% CI 44.9–63.3%] in patients with high Ki67. Results are displayed in Fig. 3.

Ki67 as a continuous variable was significantly associated with OS in multivariate analysis. Specifically, each incremental unit increase in Ki67 was associated with a 1% reduction in OS. Endocrine resistance, polymetastatic and symptomatic disease were also associated with shorter OS, whereas higher PgR was associated with extended OS. De novo disease, line of treatment, and liver metastases did not remain significant in multivariate analysis. Results are shown in Table 3. Baseline lung/pleural involvement was not prognostic for OS in univariate analysis (p = 0.950) and thus was not incorporated into multivariate analysis.

**Table 3:** Multivariate Cox regression for overall survival

Characteristics		HR*	*p*	95% CI
ECOG	1 & 2 vs. 0	3.18	< 0.001	1.96–5.15
De novo	de novo vs. rec.	1.05	0.856	0.65–1.67
Polymetastatic	> 5 mets vs. less	2.43	0.008	1.26–4.70
Line of Tx	1st vs. 2nd	0.75	0.288	0.44–1.28
ER	continuous	0.99	0.132	0.98-1.00
PgR	continuous	0.99	< 0.001	0.98–0.99
Ki67	continuous	1.01	0.007	1.00-1.03
Baseline liver inv.	yes vs. no	1.12	0.642	0.71–1.76
Endocrine resistance	yes vs. no	2.61	0.001	1.47–4.64

*Abbreviations:* *HR,* – hazard ratio, *ECOG,* – performance status assessed per Eastern Cooperative Oncology Group; rec. – reccurent disease; *Tx,* – treatment; *ER,* – estrogen receptor; *PgR,* – progresterone receptor; inv. – involvement; *HR > 1 indicates a higher hazard, whereas HR < 1 indicates a lower hazard in the first variable category

#### Subgroup Analyses by Ki67 Status for Overall Survival

**Fig. 4:**
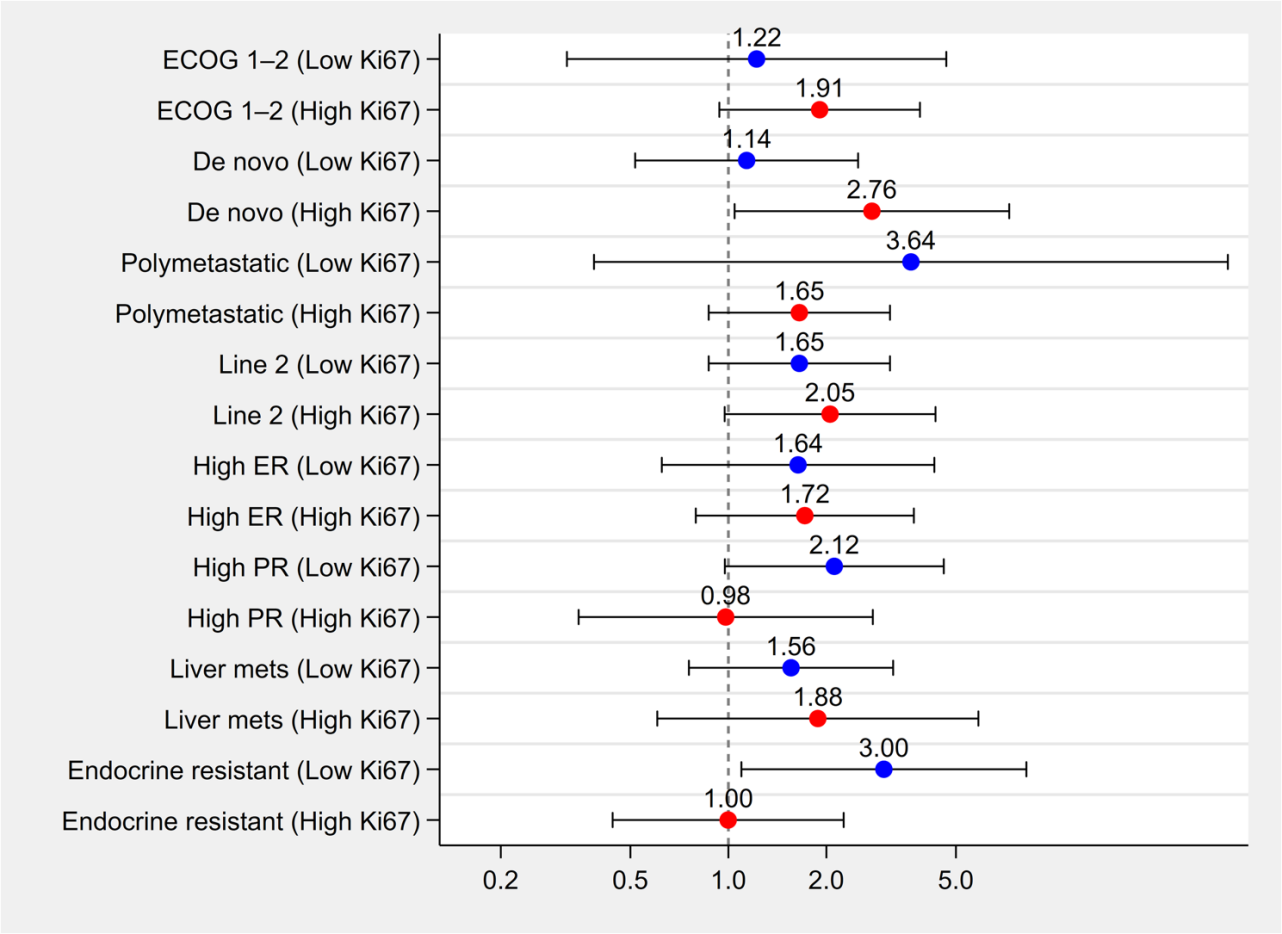
Adjusted odds ratios (ORs) for 24-month overall survival across clinical subgroups, stratified by Ki67 expression level. Each clinical subgroup (e.g., ECOG performance status, disease presentation, metastatic burden, hormone receptor status) was analyzed separately in patients with low versus high Ki67. Multivariable logistic regression models were fitted within each Ki67 stratum. Blue and red dots represent ORs for low and high Ki67 groups, respectively. Horizontal bars indicate 95% confidence intervals. A vertical dashed line at OR = 1.0 marks the null effect

To evaluate whether the prognostic impact of Ki67 on 24-month OS differed across clinical subgroups, we tested interaction terms between Ki67 (high vs. low) and key clinicopathological variables. None of the interaction terms reached statistical significance. Specifically, interaction p-values were as follows: de novo status (p = 0.165), polymetastatic disease (p = 0.507), line of treatment (p = 0.319), high ER expression (p = 0.940), high PR expression (p = 0.246), liver metastases (p = 0.783), and endocrine resistance (p = 0.096). These results suggest that the prognostic effect of Ki67 was directionally consistent across subgroups, with no significant effect modification. Results are visually summarized in Fig. 4.

#### Ki67 in Early Breast Cancer

Among the 181 patients with recurrent metastatic disease (49.2% of the study population), the median PFS was significantly shorter compared to patients with de novo metastatic breast cancer (median PFS: 19.8 months vs. not reached, *p* < 0.001).

Ki67 expression from the early breast cancer setting was available in 98 patients with recurrent disease (54.1%), all of whom had the assessment performed at our institution using standardized procedures. To ensure methodological consistency, Ki67 values obtained externally without central verification were excluded from this analysis.

Of these 98 patients, 49 had Ki67 results available only from the early breast cancer setting, while the remaining 49 had Ki67 assessed in both the early and metastatic settings. For the main analysis, the value obtained from the metastatic setting was used. In the present analysis, however, Ki67 from the early breast cancer setting was evaluated. In this subgroup, 40 tumors (40.8%) exhibited low Ki67 expression (≤ 15%), while 58 tumors (59.2%) had high Ki67 expression (> 15%). The median PFS was 19.8 months in patients with low Ki67 compared to 14.4 months in those with high Ki67; however, this difference was not statistically significant (*p* = 0.90).

### Discussion

The identification of prognostic factors for advanced breast cancer patients undergoing CDK4/6i therapy is of paramount importance. Our research has revealed that the Ki67 proliferation index serves as a prognostic marker for both PFS and OS.

Contrasting with the use of CDK4/6i in early-stage breast cancer, the significance of Ki67 in the context of metastatic breast cancer treated with CDK4/6i is not well-defined [21]. Data from small-scale retrospective studies have yielded inconsistent results; some have demonstrated a correlation between Ki67 levels and PFS [22], whereas other did not [15].

In our study, we compared patient groups characterized by low and high Ki67 expression and found them to be comparable across major clinicopathologic factors.

Interestingly, patients with low Ki67 demonstrated significantly longer PFS and OS, despite having a higher prevalence of polymetastatic disease. This seemingly paradoxical finding may be at least partially explained by fundamental differences in tumor biology between low- and high-proliferation breast cancers.

 Low Ki67 is typically associated with indolent tumor biology and better outcomes in HR–positive breast cancer [23]. Such tumors tend to present with lower tumor burden in early breast cancer [24] and are associated with significantly better prognosis compared to tumors with higher Ki67 expression [25]. These tumors are typically slower-growing and less aggressive, which may translate into more favorable outcomes and longer survival, even after distant metastasis has occurred. In contrast, high Ki67 is generally linked to higher proliferation rates, endocrine resistance, and more aggressive clinical behavior. Thus, patients with low Ki67 likely have inherently more indolent disease, leading to longer PFS and OS—even in the presence of more extensive metastatic involvement. However, this is not always straightforward, as some analyses have reported comparable survival outcomes across Ki67 strata [26].

An additional explanation, and likely the most important one, may relate to treatment effect. Patients with low Ki67 tumors, typically more endocrine-sensitive, may have derived maximum benefit from ET combined with CDK4/6 inhibition [27]. In contrast, high Ki67 tumors may exhibit partial endocrine resistance and less durable response to such therapy. Notably, PAM50-defined intrinsic subtypes have demonstrated independent prognostic value in HR+/HER2 − advanced breast cancer treated with CDK4/6i and ET [28]. In that analysis, median PFS in Luminal A patients was approximately double that observed in the Luminal B group. Moreover, Ki67 expression was significantly lower in Luminal A tumors compared to Luminal B [28]. Although genomic assays provide more comprehensive biological stratification, Ki67 appears to capture clinically relevant biological behavior in this setting, at least partially, and may serve as a pragmatic surrogate marker in routine clinical practice.

Consistent with prior studies, our findings corroborate that patients with a lower disease burden demonstrate prolonged benefit from treatment [29]. Furthermore, we have observed that patients presenting with de novo disease exhibit a longer survival compared to those with recurrent disease, aligning with previously reported results [30]. By contrast, patients with HR+/HER2 − advanced breast cancer who develop liver metastases face a significantly poorer prognosis, even with CDK4/6i treatment [31].

The Eastern Cooperative Oncology Group performance status is a recognized prognostic indicator for overall survival in metastatic breast cancer [11]. Our findings extend its significance, showing a substantial correlation with PFS as well. Unlike findings from other studies [16, 32], the CDK4/6i treatment line did not emerge as a significant prognostic factor for either PFS or OS in our research. This variance could be attributed to the fact that a predominant portion of our patient cohort received treatment in a first-line setting.

This stratified analysis highlights the potential modifying role of tumor proliferation (as measured by Ki67) on the prognostic relevance of established clinical variables. The exploratory subgroup interaction analysis for OS revealed no statistically significant interactions between Ki67 and major clinicopathological features. For PFS, the effects of ECOG performance status, polymetastatic burden, and endocrine resistance were consistent across Ki67 strata. However, the differential effect observed for de novo presentation suggests that low- and high-proliferative tumors may represent biologically distinct risk profiles. These findings support the integration of Ki67 into individualized prognostic models in this population.

The application of immunohistochemical Ki67 evaluation in routine practice remains a subject of debate due to inter-laboratory variability and dependence on institutional expertise [33]. The absence of a standardized approach to Ki67 assay performance continues to pose challenges in clinical research, particularly when dealing with real-world data. Therefore, the employment of a precise and robust assay for the reproducible detection of Ki67 is crucial [34]. The Ki67 immunohistochemistry assay utilized in our study is a validated instrument, previously employed in the monarchE clinical trial [21]. Given that our institution is the national reference center for oncology diagnostics and treatment, in cases of discrepancies between external primary results and internal re-evaluation, the result established at our center was considered definitive. Notably, a discrepancy in Ki67 assessment between the initial external result and our internal verification was observed in 17 out of 51 cases (33.3%), underscoring the critical importance of the aforementioned factors.

It may seem counterintuitive that a drug class targeting cell division, such as CDK4/6i, would be highly effective in tumors with low proliferative capacity. However, several small real-world analyses, as well as our own findings, suggest that patients with low Ki67 tumors may in fact derive superior benefit from CDK4/6i therapy [22, 35].

From a mechanistic standpoint, low Ki67 tumors are typically hormone-dependent and retain functional cell-cycle regulation. CDK4/6i, when combined with ET, induce G1-phase arrest even in low-proliferation tumors by targeting the subset of cells that enter the cell cycle [36]. Although these tumors are indolent, they continue to exhibit low-level proliferative activity, which is effectively suppressed by CDK4/6 inhibition, resulting in durable disease control [28].

Moreover, CDK4/6i may exert additional biological effects beyond direct cell-cycle arrest, including induction of cellular senescence [37], modulation of the tumor immune microenvironment [38], and interference with endocrine resistance pathways. These broader mechanisms could further contribute to the clinical benefit observed in tumors with low proliferation rates.

While our study provides valuable insights, it is important to acknowledge its limitations, the most significant being its retrospective nature. The Ki67 cutoff identified for optimal prognostic significance in our analysis was 15%. This threshold aligns with the stratification used in the Penelope B trial, which also applied a 15% Ki67 cutoff to assess palbociclib as an adjuvant treatment for residual high-risk early breast cancer [39].

However, recommendations by the International Ki67 in Breast Cancer Working Group and the 17th St Gallen International Breast Cancer Conference propose using Ki67 expression cutoff of either 5% or less, or 30% or more, for clinical decision-making in early breast cancer [4, 40]. Given that Ki67 is a continuous biomarker, the determination of risk cutoffs is subject to ongoing discussion and requires further validation. Nevertheless, we used the dichotomized value of Ki67 only for Kaplan-Meier estimates, while continuous values of Ki67 were incorporated for proportional hazards regression models. Guidelines from the European Group on Tumor Markers regarding Ki67 have highlighted the limited research in the metastatic setting and the discrepancies in the use of various cutoff points. They recommended further research to establish an optimal cutoff point or to evaluate the use of Ki67 as a continuous variable [41]. From a biological perspective, Ki67 should be regarded a continuous variable [42]. Thus, interpreting Ki67 values close to a cutoff point requires careful consideration since these cases may exhibit comparable biological behavior [42].

Breast cancer’s biological characteristics can evolve over time, leading to discrepancies between primary tumors and asynchronously paired metastases [43]. Consequently, Ki67 expression may vary when assessed during initial diagnosis of primary breast cancer and at the time of disease recurrence in metastatic lesions [44, 45]. In some studies, higher levels of Ki67 immunohistochemical expression were observed in metastases compared to primary breast tumors assessed in the early setting [46, 47]. In contrast, other studies found lower Ki67 levels in metastases or reported similar rates of changes from high to low expression and from low to high Ki67 expression [44, 45]. Additionally, no differences in Ki67 expression were observed with regard to the site of biopsy and the time to biopsy [43]. These findings underscore the dynamic nature of breast cancer biology and the necessity for re-evaluating Ki67 expression during different stages of the disease to achieve adequate results. In the vast majority of patients in our study we assessed Ki67 in the tissue obtained in the metastatic setting.

When Ki67 was assessed from tumor samples obtained during the early breast cancer setting, no statistically significant difference in PFS was observed between patients with low and high Ki67. While the median PFS was numerically longer in the low Ki67 group (19.8 vs. 14.4 months), the lack of statistical significance may be partially attributed to the smaller sample size available for this subgroup. Another important consideration is the potential biological divergence between early and metastatic disease. In ER–positive, HER2-negative breast cancer, late recurrence is common—sometimes occurring more than two decades after initial treatment. Tumor biology, including proliferative capacity, may evolve during this interval due to clonal selection, treatment pressure, or tumor dormancy. As a result, Ki67 expression assessed at initial diagnosis may not accurately reflect the current biological behavior of the tumor at the time of metastatic relapse. Therefore, Ki67 evaluation performed shortly before initiating treatment in the metastatic setting may provide a more relevant prognostic and predictive signal, as appears to be the case in our study.

It is also worth noting that Ki67 assessment in early breast cancer was not routinely performed in our cancer center prior to the 2013 St. Gallen International Consensus, which was the first to formally introduce clinico-pathologic surrogate definitions of intrinsic breast cancer subtypes based on markers such as ER, PR, HER2, and Ki67 [23]. Consequently, most early breast cancer samples obtained before 2013 at our institution lack Ki67 assessment.

Updated recommendations from the International Ki67 in Breast Cancer Working Group emphasize the importance of preanalytical handling, and participation in quality control programs to maintain analytical validity [4]. In our study, samples were not sent for external, central validation, which is a limitation. However, the vast majority of samples were either derived or verified at our center. We observed some discrepancies in Ki67 verification, with assessments at our center differing from primary assessments conducted outside, roughly splitting evenly between higher and lower Ki67 values. Given our expertise as a tertiary referral hospital and national research institute, coupled with our participation in external quality control assessments, the results of our Ki67 assessments can be considered reliable. Additionally, our dedicated team of breast pathologists further reduces the potential for interobserver interpretation error. Nonetheless, despite being a national reference center for oncology diagnostics and treatment, the lack of external Ki67 validation may be perceived as a limitation of the study.

The quest for new biomarkers that can accurately differentiate between patients likely to experience a prolonged response to treatment and those at risk of rapid disease progression remains a pivotal clinical challenge. Currently, the landscape of molecular markers for CDK4/6i resistance is sparse. In approximately 44% of cases, potential biomarkers indicative of resistance remain elusive within circulating tumor DNA [48]. Beyond the estrogen receptor and human epidermal growth factor receptor 2, the repertoire of biomarkers guiding therapeutic decisions for CDK4/6i in metastatic breast cancer is limited [8]. In an exploratory analysis of tumor samples from the phase III MONALEESA-2, MONALEESA-3, and MONALEESA-7 trials, PAM50-based intrinsic breast cancer subtypes—specifically luminal A, luminal B, normal-like, HER2-enriched, and basal-like—showed a significant association with PFS [49]. Notably, all subtypes except basal-like demonstrated a substantial PFS benefit when ribociclib was added to endocrine therapy. Despite providing less extensive data, Ki67 assessment remains widely available and cost-effective compared to the available multianalyte signatures, and can be used in combination with other prognostic factors [41].

Our findings suggest that Ki67 may have the potential to be an informative biomarker in this context. However, further research is essential to validate whether Ki67 can be reliably integrated into the existing framework of biomarkers for informing treatment strategies with CDK4/6i.

## Conclusions

Ki67 appears to be a prognostic factor in advanced breast cancer patients treated with CDK4/6 inhibitors. Patients with low Ki67 had the most favorable prognosis.

## Electronic Supplementary Material

Below is the link to the electronic supplementary material.

Updated References with Mendeley Cite

## Data Availability

The datasets used and/or analysed during the current study are available from the corresponding author upon reasonable request.

## Abbreviations

CDK4/6 inhibitors: cyclin–dependent kinase 4/6 inhibitors
ECOG: Eastern Cooperative Oncology Group
ER: estrogen receptor
ET: endocrine therapy
HER2: human epidermal growth factor receptor 2
HR: hormone receptor
IHC: immunohistochemistry
NordiQC: Nordic Immunohistochemical Quality Control
OS: overall survival
PFS: progression–free survival
PgR: progesterone receptor
PS: performance status
ROC: receiver operating characteristic
